# Leber congenital amaurosis/early-onset severe retinal dystrophy: clinical features, molecular genetics and therapeutic interventions

**DOI:** 10.1136/bjophthalmol-2016-309975

**Published:** 2017-07-08

**Authors:** Neruban Kumaran, Anthony T Moore, Richard G Weleber, Michel Michaelides

**Affiliations:** 1 UCL Institute of Ophthalmology, University College London, London, UK; 2 Moorfields Eye Hospital NHS Foundation Trust, London, UK; 3 University of California San Francisco, San Francisco CA, California, USA; 4 Casey Eye Institute, Oregon Health and Science University, Portland, Oregon, USA

**Keywords:** (MeSH terms): retinal diseases, leber congenital amaurosis, lca, early onset severe retinal dystrophy, eosrd, severe early childhood onset retinal dystrophy, secord, gene therapy, rtinal rod photoreceptor cells, retinal cone photoreceptor cells, review

## Abstract

Leber congenital amaurosis (LCA) and early-onset severe retinal dystrophy (EOSRD) are both genetically and phenotypically heterogeneous, and characterised clinically by severe congenital/early infancy visual loss, nystagmus, amaurotic pupils and markedly reduced/absent full-field electroretinograms. The vast genetic heterogeneity of inherited retinal disease has been established over the last 10 - 20 years, with disease-causing variants identified in 25 genes to date associated with LCA/EOSRD, accounting for 70–80% of cases, with thereby more genes yet to be identified. There is now far greater understanding of the structural and functional associations seen in the various LCA/EOSRD genotypes. Subsequent development/characterisation of LCA/EOSRD animal models has shed light on the underlying pathogenesis and allowed the demonstration of successful rescue with gene replacement therapy and pharmacological intervention in multiple models. These advancements have culminated in more than 12 completed, ongoing and anticipated phase I/II and phase III gene therapy and pharmacological human clinical trials. This review describes the clinical and genetic characteristics of LCA/EOSRD and the differential diagnoses to be considered. We discuss in further detail the diagnostic clinical features, pathophysiology, animal models and human treatment studies and trials, in the more common genetic subtypes and/or those closest to intervention.

## Introduction

Inherited retinal disease (IRD) represents the second most common cause of legal blindness in childhood and the leading cause among the working aged population in England and Wales.^1^ Prior to identification of the causative genes, clinicians classified patients into groups based on Mendelian inheritance, age of onset and clinical features. The discovery of the underlying genetic causes over the last two decades has led to far greater understanding of disease mechanisms in IRD which has informed development of novel therapies, culminating in the first gene therapy trials for RPE65-associated Leber congenital amaurosis (LCA)/Early Onset Severe Retinal Dystrophy (EOSRD) in 2008.^2–4^

LCA was first described by Theodore Leber in 1869 and is now used to describe a group of severe recessively inherited, early infantile onset rod–cone dystrophies.^5^ In 1916, Leber described what he considered a milder form of the same disease^6^ which has had several names, including EOSRD, severe early childhood-onset retinal dystrophy (SECORD)^7^ and early-onset retinitis pigmentosa. Whereas LCA is congenital or presents within the first few months of life, is associated with nystagmus, poor pupil responses and in most instances an undetectable full-field electroretinogram (ERG); EOSRD/SECORD is defined as a severe retinal dystrophy presenting after infancy and usually before the age of 5 years. Other distinguishing features of EOSRD/SECORD include better residual visual function and small ERG signals, particularly for the lesser-affected photoreceptor system. Of note, there is significant overlap between the molecular causes of LCA and EOSRD, with some genes causing both clinical phenotypes. However, certain genes are more frequently associated with LCA, for example, *GUCY2D*, *NMNAT1*, *CEP290* and *AIPL1*, whereas mutations in others including *RPE65*, *LRAT* and *RDH12*, more commonly result in an EOSRD phenotype.

**Figure 1:**
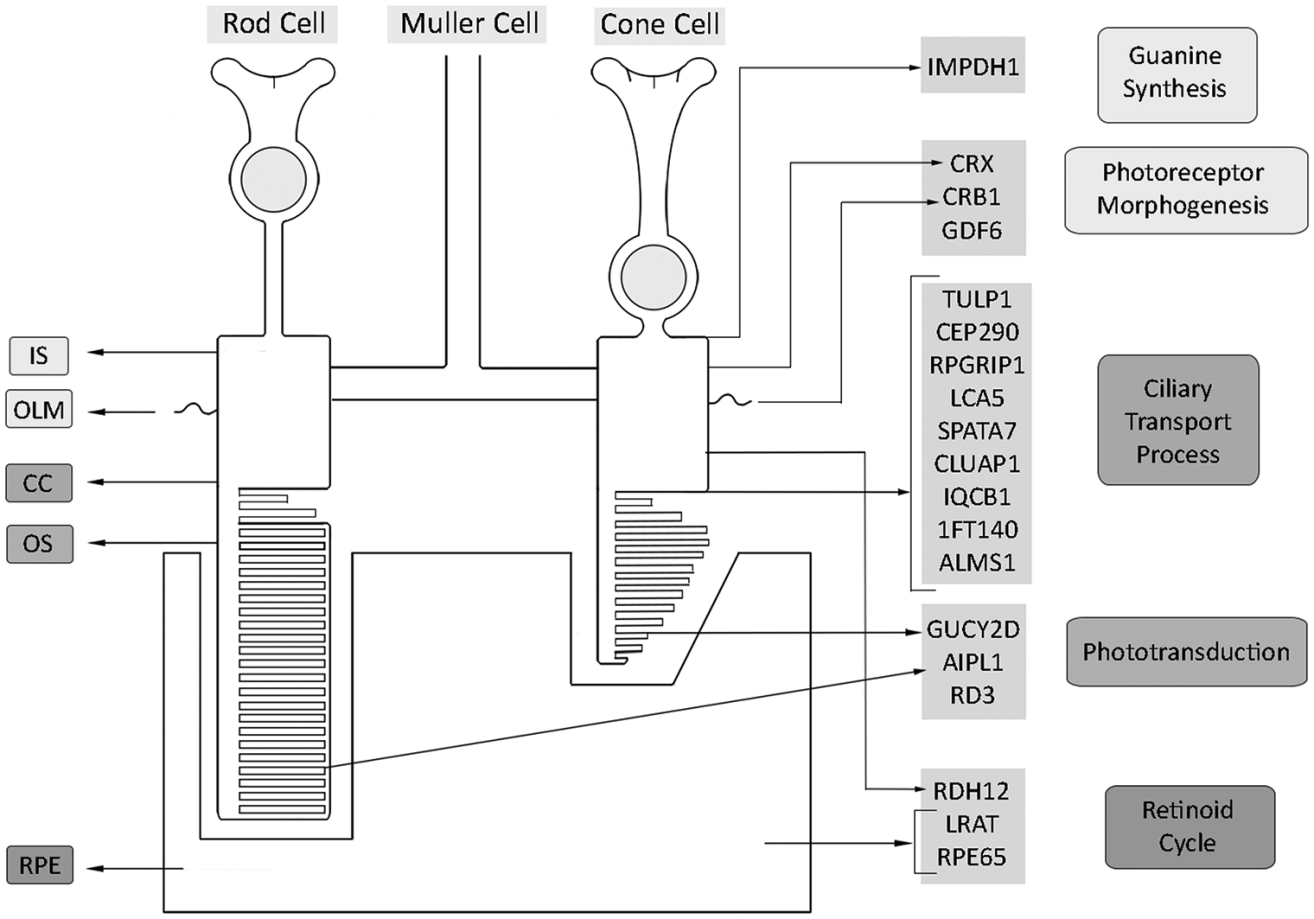
Spatial representation of expression of LCA/EOSRD genes, grouped according to their proposed function. EOSRD, early-onset severe retinal dystrophy; LCA, Leber congenital amaurosis.

To date, mutations in 25 genes have been identified as causing LCA/EOSRD; most are expressed solely or predominantly in the retina or the retinal pigment epithelium (RPE).^8^ These genes have been shown to encode proteins with a diverse range of retinal functions, including phototransduction, the visual cycle and photoreceptor development/integrity (table 1, figure 1).^9–11^

**Table 1:** Overview of the genes associated with LCA/EOSRD, the encoded proteins, their proposed function(s) and estimated frequency

Locus name	Causative gene	Protein	Protein function(s)	Approximate frequency
LCA 1	*GUCY2D*∗	Guanylate cyclase-1	Phototransduction	10%–20%
LCA 2	*RPE65*†	Retinoid isomerase	Retinoid cycle	5%–10%
LCA 3	*SPATA7*	Spermatogenesis-associated protein 7	Photoreceptor ciliary transport	3%
LCA 4	*AIPL1*∗	Aryl-hydrocarbon-interacting-protein-like 1	Phototransduction/protein biosynthesis	<5%
LCA 5	*LCA5*	Libercilin	Photoreceptor ciliary transport	1%–2%
LCA 6	*RPGRIP1*	Retinitis pigmentosa GTPase regulator-interacting protein 1	Photoreceptor ciliary transport	5%
LCA 7	*CRX*	Cone–rod homeobox	Photoreceptor morphogenesis	1%
LCA 8	*CRB1*∗	Crumbs homologue 1	Photoreceptor morphogenesis	10%
LCA 9	*NMNAT1*∗	Nicotinamide nucleotide adenyltransferase1	Coenzyme NAD biosynthesis	Unknown
LCA 10	*CEP290*	Centrosomal protein 290 kDA	Photoreceptor ciliary transport	15%–20%
LCA 11	*IMPDH1*	Inosine 5’—monophosphate dehydrogenase 1	Guanine synthesis	5%
LCA 12	*RD3*	Protein RD3	Protein trafficking	<1%
LCA 13	*RDH12*†	Retinol dehydrogenase 12	Retinoid cycle	10%
LCA 14	*LRAT*†	Lecithin:retinol acyl transferase	Retinoid cycle	<1%
LCA 15	*TULP1*	Tubby-like protein	Photoreceptor ciliary transport	<1%
LCA 16	*KCNJ13*	Kir7 inwardly rectifying potassium channel	Phototransduction	Unknown
LCA 17	*GDF6*	Growth differentiation factor 6	Photoreceptor morphogenesis	Unknown
	*OTX2*	Orthodenticle homeobox 2 protein	Photoreceptor differentiation	Unknown
	*CABP4*	Calcium-binding protein 4	Phototransduction	Unknown
	*CLUAP1*	Clusterin associated protein 1	Photoreceptor ciliary transport	Unknown
	*IQCB1*	IQ motif containing B1 protein	Photoreceptor ciliary transport	Unknown
	*DTHD1*	Death-domain containing protein 1	Unknown	Unknown
	*IFT140*	Intraflagellar transport 140 chlamydomonas homologue protein	Photoreceptor ciliary transport	Unknown
	*ALMS1*	ALMS Protein	Photoreceptor ciliary transport	Unknown
	*PRPH2*	Perpherin	Photoreceptor outer segment structure/stabilisation	Unknown

*Genes associated with EOSRD.

†Genes more frequently associated with LCA.

Other genes have shown no clear predilection.

EOSRD, early-onset severe retinal dystrophy; LCA, Leber congenital amaurosis.

Given recent advances in understanding of the molecular basis of these disorders and the ongoing clinical trials of novel therapies, we herein review the clinical characteristics, animal models and pathophysiology of LCA/EOSRD, prioritising the more common genotypes and/or those closest to intervention.

### Clinical characteristics

LCA/EOSRD has a prevalence between 1 in 33 000^12^ to 1 in 81 000,^13^ and is believed to account for ≥5% of all IRD.^12^

**Figure 2:**
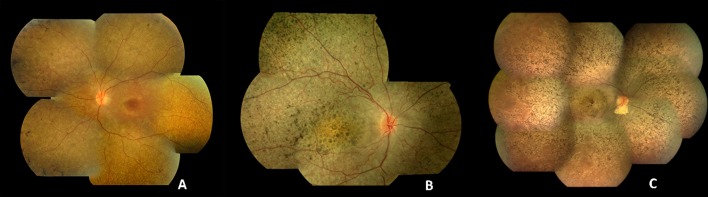
Colour fundus photographs of patients with recognisable LCA/EOSRD clinical phenotypes. (A) *RPE65*-retinopathy, associated with a blonde fundus, peripheral, white punctate lesions and normal central macular appearance in keeping with central preservation of outer retina. (B) *CRB1*-retinopathy characterised by nummular pigmentation, periarteriolar sparing of the RPE and macular atrophy. (C) *RDH12*-retinopathy with characteristic dense intraretinal pigmentation and macular atrophy with pigmentation and yellowing. EOSRD, early-onset severe retinal dystrophy; LCA, Leber congenital amaurosis; RPE, retinal pigment epithelium.

LCA is associated with severe visual impairment from birth or the first few months of life accompanied by roving eye movements or nystagmus and poor pupillary light responses. Eye poking, the ‘oculodigital’ sign, is common. The ERG is undetectable or severely abnormal. Fundus examination may be normal at presentation, but a variety of abnormal fundus appearances may be present or develop over time, including disc pallor, vessel attenuation or mild peripheral pigmentary retinopathy. There may also be disc drusen, optic disc oedema or pseudopapilloedema, a flecked retina, maculopathy or nummular pigmentation (figure 2). Affected infants often have high hyperopia, or less commonly high myopia, suggesting impaired emmetropisation.^14^

The rate of loss of visual function and early childhood visual acuity vary markedly in patients with LCA/EOSRD, with certain genotypes (eg, *GUCY2D* and *AIPL1*-LCA) known to be more severe than others, with earlier more profound visual loss. Although visual outcome is variable, vision when the child is old enough to be reliably tested is in the region of 3/60 to perception of light. Given the often severe and early visual loss, other areas of development including speech, social skills and behaviour may be delayed; early involvement of a specialist paediatrician with expertise in the developmental needs of children with visual impairment can significantly mitigate/delay disruption to these other fundamental areas of childhood development.^15^

The identified genes account for approximately 70%–80% of LCA/EOSRD cases (table 1, figure 1 and below), with *GUCY2D*, *CEP290*, *CRB1*, *RDH12* and *RPE65* being the most common.^9 16–19^ It has been possible to identify certain characteristic associated phenotypes (figure 2 and below): *RDH12*-associated disease, which gives rise to an EOSRD phenotype is characterised by early-dense intraretinal pigment migration pigmentation and maculopathy. *CRB1*-associated disease has nummular pigmentation, maculopathy, relative preservation of para-arteriolar RPE, with retinal thickening and loss of lamination on optical coherence tomography. *TULP1*, *AIPL1* and *NMNAT1*-associated disease are also characterised by maculopathy which in the case of patients with *NMNAT1* mutations is of early onset and extensive.

Most cases of LCA/EOSRD occur in otherwise normal infants and any non-ocular symptoms or signs should be investigated for syndromic retinal dystrophies or neurometabolic disease, usually in conjunction with a paediatrician. Renal involvement (nephronophthisis which can lead to end-stage renal failure) may be seen in some genetic subtypes of LCA/EOSRD (eg, *IQCB1*, *IFT140* and *CEP290*) as part of syndromes including Senior-Loken Syndrome and Joubert Syndrome.^20–22^ Early molecular diagnosis can help prioritise which children should have ongoing systemic investigations.

The differential diagnosis of an infant with severe visual loss and nystagmus is broad and includes both syndromic and non-syndromic diseases (table 2). Conditions that are important to be distinguished from LCA/EOSRD include complete and incomplete Achromatopsia, S-cone monochromatism, complete and incomplete congenital stationary night blindness and albinism; however careful examination and detailed investigation including electrophysiology usually allow an accurate diagnosis to be established. Molecular genetic testing may then allow a more specific diagnosis (table 2).

**Table 2:** Selected conditions/syndromes to be considered in the differential diagnosis of isolated LCA/EOSRD

Condition/syndrome	Assessments and features that assist in distinguishing from LCA/EOSRD
Achromatopsia	Achromatopsia is characterised by absent/markedly reduced cone responses with normal rod ERG responses and a stationary natural history; compared with the flat/markedly reduced full-field ERGs and progressive degeneration seen in LCA/EOSRD.
Congenital stationary night blindness	Can be differentiated on the basis of ERG phenotype and natural history.^94^
Ocular and oculocutaneous albinism	Clinical examination (skin, hair, eyebrows/eyelashes; iris and retina), retinal imaging (OCT and FAF) and electrophysiological assessment.
Optic nerve hypoplasia	Clinical examination and electrophysiological assessment. Of note, absent pupil reflexes suggests severe optic nerve hypoplasia rather than LCA.
Batten disease	Infantile Neuronal Ceroid Lipofuscinosis (NCL) presents with congenital or early- onset blindness, with onset by age 6 months; whereas patients with Late and Juvenile onset NCL present at 2–4 and 6 years of age or older, respectively. Patients with NCL have neurocognitive decline and can have epilepsy.
Joubert syndrome	Patients present with severe visual impairment, ocular motor abnormalities, often have the ‘molar tooth’ sign on MRI and develop nephronophthisis in later childhood.
Peroxisomal diseases	Associated features include sensorineural deafness, dysmorphic features, developmental delay, hepatomegaly and early death.
Alstrom syndrome	Presents with infantile onset nystagmus, photophobia and a cone–rod dystrophy on electrophysiological assessment. Other systemic features include childhood obesity, hyperinsulinaemia, type 2 diabetes mellitus, hepatic dysfunction, heart failure, sensorineural hearing loss and renal failure.
Cobalamin C deficiency	Has a variable phenotype with severely affected individuals showing progressive, infantile onset, metabolic, neurological and ophthalmic manifestations, including infantile nystagmus, bulls-eye maculopathy and reduced responses on ERG.

EOSRD, early-onset severe retinal dystrophy; FAF, fundus autofluorescence; LCA, Leber congenital amaurosis; OCT, optical coherence tomography.

### Selected specific genotypes

#### *GUCY2D*-associated LCA

*GUCY2D* was the first gene to be associated with LCA/EOSRD and is one of the most common, accounting for approximately 10%–20% of cases.^16^ *GUCY2D* is known to encode retinal guanylate cyclase-1 (RetGC1), expressed in photoreceptor outer segments, to a greater degree in cones than rods.^23 24^ RetGC1 is known to play an important role in photoreceptor recovery following phototransduction, thereby disease-causing variants in *GUCY2D* and subsequent RetGC1 deficiency result in the biochemical equivalent of chronic light exposure.^16^

Patients with *GUCY2D*-associated LCA often have relatively normal fundi, and also experience significant photophobia in contrast to most other LCA/EOSRD genotypes. There can be relatively preserved outer retinal / photoreceptor structure on optical coherence tomography (OCT) in many patients, although foveal cone outer segment abnormalities and foveal cone loss has been observed.^25 26^ In contrast to other forms of LCA/EOSRD which have a rod–cone dystrophy phenotype, in *GUCY2D* disease patients often retain substantial rod function, with a smaller subset having detectable but reduced cone function (ie, cone–rod disease), based on ERG, psychophysical assessments and vision-guided mobility testing.^25^ Due to the majority of patients lacking cone-mediated vision, patients often present with markedly reduced visual acuity and a lack of colour perception.^27^

Disease mechanisms and interventional approaches have been explored in several animal models of *GUCY2D*-LCA. Initially, gene replacement was investigated using an HIV1-based lentiviral vector in a naturally occurring chicken model and showed improved optokinetic reflexes and volitional visual behaviour.^28^ Subsequently, multiple groups have had therapeutic success in engineered mouse models. Both adeno-associated virus serotype 5 (AAV5) and recombinant adeno-associated virus serotype 2/8 (AAV 2/8) vectors carrying animal and human complementary DNA (cDNA), respectively, have been shown to rescue retinal function and preserve cone photoreceptors in RetGC1 knock-out mouse models.^29–31^ Moreover, an AAV8 based-gene replacement therapy, using the tyrosine capsid mutant AAV8(Y733F), has been shown to restore both useful cone and rod-mediated vision in a RetGC1/RetGC2 double knock-out mouse.^32^ These successful animal studies combined with the relative outer retinal preservation observed in patients with *GUCY2D*-LCA has resulted in advanced preparation for a human gene therapy trial.

#### *RPE65*-associated LCA

*RPE65*-associated LCA/EOSRD accounts for approximately 5%–10% of cases and has been the subject of the most clinical trials to date.^9^ *RPE65* encodes a 65kD retinoid isomerase expressed in the RPE; a critical component of the retinoid (visual) cycle.^33 34^ As such, patients with *RPE65* deficiency have a lack of 11-*cis*-retinal. Most children with RPE65 deficiency have an EOSRD phenotype with profound night blindness from birth but residual cone-mediated vision and often mild if any nystagmus. It has been suggested that cone photoreceptors have an alternative supply of 11-*cis*-retinal independent of RPE65, thus allowing cone-mediated vision in younger patients.^35^ However, due to the 11-*cis*-retinal deficiency in rod photoreceptors there is early and profound nyctalopia.

OCT studies have demonstrated relatively normal retinal thickness in some patients; with more commonly a central macular area of relatively preserved retina with a ring of thinning or more widespread retinal loss. There appears to be no clear relationship between age and thinning in the first three decades.^36 37^ This phenotypic variability irrespective of age, and varying both between and within families, is typical of the marked heterogeneity characterising IRD as a whole. *RPE65*-deficiency is also associated with reduced or absent autofluorescence on fundus autofluorescence imaging, suggesting low or absent levels of lipofuscin in the RPE.^38^

In 2001, Acland *et al* described successful gene therapy in Briard dogs with *RPE65*-retinopathy using subretinal delivery of recombinant adeno-associated virus serotype 2 (AAV2) vector with canine *RPE65* cDNA under the control of the cytomegalovirus chicken beta actin promoter.^39^ Injected eyes of these dogs showed improved ERG, pupillometry and flash evoked cortical potentials in the dark-adapted state. Furthermore, these improvements were sustained for over 10 years.^39 40^ Subsequently, murine models of *RPE65* deficiency have been characterised and rescued using gene replacement therapy, with significant improvement in visual function in both *RPE65* ^-/-^ mice and naturally occurring *RPE65* mutant mice (*Rd12* mouse) and the demonstration of a dose–response relationship.^36 41–44^

Success in canine and murine models^39 45^ resulted in four phase 1 gene therapy clinical trials in humans in close succession.^2–4 46^ The 2 to 5-year follow-up data on all four trials has been very encouraging, showing the intervention to be safe with varying levels of efficacy noted; however, most studies at later intervals have shown a lack of durability of the improvements, with continuing retinal degeneration even in patients with improved retinal function.^46–49^ Of the 12 patients enrolled in the UK trial, an initial improvement in retinal sensitivity, dark-adapted perimetry and vision-guided mobility was noted in six, five and three patients, respectively. This improvement was not sustained, with only two patients having residual improvement greater than preinterventional levels, 3  years following subretinal injection.^47^ Similar results were noted in the other three clinical trials, with Testa *et al* reporting sustained improvement in visual acuity, visual field, nystagmus frequency and pupillometry at 3 years.^48^ Weleber *et al* noted variable improvement in one or more parameters of visual function in 9 of 12 patients.^46^ Jacobson *et al* described visual function improved in all 15 patients, to different degrees, with improvements being localised to treated areas, but a waning of improvement over time and continued retinal degeneration.^49^ It has been proposed that continued retinal degeneration may be due to suboptimal *RPE65* expression in the human retina, resulting in subsequent work to optimise the vector, promoter and transgene hoping to result in more durable benefit.^47 50^

Acknowledging the known impaired structural properties of the visual pathway observed in visually deprived animals^51^ and blind human studies,^52^ Ashtari *et al* investigated the plasticity of the visual system following retinal gene therapy in patients with *RPE65*-LCA using non-invasive multimodal neuroimaging.^53^ They reported improvements and normalisation in the visual pathway corresponding to the retinal injection areas in the treated eyes, which were maintained over time, compared with the visual pathway for the untreated eyes which continued to deteriorate. This would support the belief that early intervention would be most likely to result in the greatest benefit. However, of note the 10 subjects in this study were aged 9 to 45 years, suggesting that intervention in adulthood could still be beneficial.

Furthermore, recently, one phase III trial (NCT00999609) and a phase I/II trial (NCT01496040) have been completed with another two  phase I/II trials (clinicaltrials.gov identifiers: NCT02781480, NCT00821340,) now underway.

Pharmacological intervention has also been investigated to potentially overcome the biochemical blockade, following successful rescue in animal models.^54 55^ A phase I/II clinical trial examining the efficacy of the synthetic prodrug QLT091001, a precursor to 9-*cis*-retinal, has been conducted in 18 patients (13 with *RPE65*-LCA and five with *LRAT*-LCA—as both encoded proteins participate in the retinoid cycle (figure 1)).^56^ Patients received a once-daily oral dose of 40 mg/m^2^/day for 7 consecutive days. Following this, 44% of patients had a >20% increase in isopter area in one or both eyes, as assessed by Goldmann visual field testing, and 67% of patients showed a>5 ETDRS letter score increase in one or both eyes.^56^ A phase III trial is due to commence in the near future.

#### *AIPL1*-associated LCA

*AIPL1* encodes Aryl-hydrocarbon-interacting-protein-like 1—a photoreceptor-specific cochaperone that interacts specifically with the molecular chaperone HSP90 to modulate the stability of and assembly of the HSP90 substrate, retinal cGMP phosphodiesterase.^57 58^

Sequence variants in *AIPL1* are associated with a relatively severe congenital LCA phenotype, with severe and rapidly progressive visual loss, accounting for less than 5% of LCA.^9 59 60^ Aboshiha *et al* investigated a worldwide cohort of 42 molecularly confirmed patients, collecting data on demographics, visual acuity, fundus examination, OCT findings and genotype. This study identified p.W278X (c.834G>A) as the most common *AIPL1* variant, occurring in at least one allele in 62% of patients.^61^ Interestingly, hand-held OCT imaging identified four patients (three of whom harboured the aforementioned common variant) with relative preservation of central outer retinal structure, all of whom were younger than 4 years of age.^61^

Gene replacement therapy using an AAV2/8 vector in an *AIPL1* knock-out mouse model has been shown to result in restoration of cellular function with photoreceptor cell preservation and improved retinal function, despite the severe rapidly progressive early-onset retinal degeneration seen in this mouse model.^62^ The early visual loss to perception of light or worse, the high-resolution OCT evidence of retained outer retinal structure early in life and the successfully treated animal models, raise the possibility of a gene therapy-based approach in *AIPL1*-LCA early in life; with a human clinical interventional study being in the advanced stages of development.

#### *RPGRIP1*-associated LCA

*RPGRIP1*-LCA is believed to be responsible for approximately 5% of cases.^63 64^ RPGRIP1 is a binding partner of retinitis pigmentosa GTPase regulator (RPGR) and is essential for anchoring RPGR at the connecting cilia between the inner and outer segments of photoreceptor cells.^65–69^

In contrast to other forms of LCA, *RPGRIP1*-LCA appears to be relatively non-progressive, following an initial rapid decline in visual function.^18^ Furthermore, photoreceptors in the central retina appear to remain present for a significant period following deterioration of visual function.^70^ These features suggest a wide window of opportunity for potential intervention.

An *RPGRIP1* knock-out mouse and *RPGRIP1* deficient mouse and dog models have been characterised and have provided models for preclinical gene replacement studies.^55–57^ In *RPGRIP1* knock-out mice, both improved outer segment morphology and photoreceptor survival, and better preservation of ERG responses have been demonstrated with *RPGRIP1* gene replacement using both AAV2 and AAV8 vectors.^71 72^ Improved photoreceptor function up to 24 months postsubretinal injection has also been shown in the canine model.^73^

#### *CRB1*-associated LCA

Disease-causing sequence variants in *CRB1* have been identified in a broad range of phenotypes, including the early-onset disorders LCA/EOSRD and retinitis pigmentosa with and without a Coats-like vasculopathy, a later-onset macular dystrophy and isolated autosomal recessive foveal retinoschisis.^74–77^ Approximately 10% of LCA/EOSRD patients harbour variants in *CRB1*.^9 18^ The CRB1 protein is known to colocalise with the zonula adherens, forming a major component of the outer limiting membrane and is believed to have a role in retinal development.

Both the severity and rate of progression vary significantly between patients with *CRB1*-associated LCA/EOSRD, with clinical examination and OCT findings often allowing directed molecular screening of *CRB1*.^74 75 78^ Characteristic findings include macular atrophy, nummular pigmentation, relative para-arteriolar preservation of the RPE and retinal thickening with loss of lamination—in direct contrast to other forms of LCA/EOSRD where progressive retinal thinning is commonplace.

#### *CEP290*-associated LCA

*CEP290*-LCA/EOSRD accounts for 15%–20% of cases, thereby representing one of the most common genetic causes.^17^ CEP290 localises to the centromeres and the connecting cilia of photoreceptors. The intronic variant c.2991+1655 A>G is the most common disease-causing mutation (especially in European countries and in the USA), having been identified in at least one allele in 77% of patients (n=43) in a study of *CEP290*-LCA.^79^

Retinal examination can be relatively normal in infancy with significant variability in visual function, with no correlation between age and visual acuity (VA) noted over an average follow-up period of 10 years; although severe VA loss (counting fingers or worse) is seen in most, but not all, children in the first decade.^17 79 80^

Interestingly, OCT studies have shown that despite profound cone dysfunction, the foveal outer nuclear layer (cone nuclei) is structurally detectable until the fourth decade of life in some patients, although with abnormal inner and outer segments in contrast to the early loss of rod photoreceptors.^81^ These findings suggest a potential window of opportunity—wider for possible cone rescue than rod. Gene therapy-based intervention has been explored in vitro with a lentiviral vector containing human *CEP290* and been shown to effectively transduce patient-specific induced pluripotent stem cell-derived photoreceptor precursor cells and rescue the cellular phenotype.^82^ Other molecular therapeutic avenues being explored focus on the common deep intronic *CEP290* sequence variant, which creates a strong splice donor site that leads to the insertion of a cryptic exon encoding a premature stop codon. Several approaches have been suggested, including the use of antisense oligonucleotide-mediated exon skipping to abrogate the disease-causing variant or correction of the splice defect using CRISPR/Cas9-mediated gene editing.^83 84^ Both of these novel approaches show promise and human clinical trials are anticipated in the near future.^85^

#### *RDH12*-associated LCA

Disease-causing sequence variants in *RDH12* are identified in up to approximately 10% of LCA/EOSRD patients.^9 19^ *RDH12* encodes retinol dehydrogenase 12, a component of the visual cycle, which when deficient is believed to result in retinal toxicity secondary to all-*trans*-retinal accumulation.^86^

Most patients with biallelic *RDH12* mutations have an EOSRD rather than LCA phenotype. There is a recognisable fundus phenotype with widespread RPE and retinal atrophy and minimal intraretinal pigmentation in early childhood, with dense intraretinal bone-spicule pigmentation developing over time (figure 2).^19^ There is early progressive macular atrophy, with pigmentation and yellowing and corresponding macular excavation on OCT and loss of fundus autofluorescence.^19^

Promising results of AAV2/8-vector-mediated *RDH12* gene replacement therapy in *Rdh12* knock-out mice has raised the possibility of human clinical trials in the future.^87^

#### *LRAT*-associated LCA

*LRAT* encodes Lecithin:retinol acyl transferase, a key enzyme in the vitamin A recycling pathway (visual cycle), with LRAT deficiency associated with a similar phenotype to RPE65-deficiency, although being far less common.^9 88^ As described for RPE65-deficiency, the synthetic prodrug QLT091001, a precursor to 9-*cis*-retinal, has been shown in a phase I trial to improve VA and kinetic visual fields in *LRAT*-LCA, with a phase III trial anticipated in the future.

### Therapeutic advances potentially applicable to advanced LCA

Stem cell therapies hold great promise for the future to restore lost retinal cells in advanced disorders, including potentially LCA. At present, human stem cell therapy has primarily been confined to RPE transplantation, with two phase I/II studies reporting safe transplantation of human embryonic stem cell-derived RPE and induced pluripotent stem cell-derived RPE, respectively.^89 90^ However, transplantation of other retinal cells, including photoreceptors—which would be needed in LCA, remains at a preclinical stage.

Clinical trials have also been conducted to investigate the therapeutic potential of electronic retinal prostheses in advanced retinal degeneration including choroideremia and retinitis pigmentosa. At present, current epiretinal or subretinal devices cannot sufficiently replace the high density of photoreceptors at the fovea and have thereby resulted in very low levels of VA improvement in a minority of patients, with a varied safety profile.^91–93^

## Conclusions

Improvements in molecular genetic testing and clinical assessments (primarily quantitative high-resolution retinal imaging) have led to a greater understanding of genotype–phenotype correlations and structure–function associations in LCA/EOSRD. This coupled with advances in gene therapy in both animal model studies and human clinical trials have resulted in a current new era of increasing clinical trials for multiple genetic subtypes of LCA/EOSRD and the cautious optimism for the development of proven successful and durable treatments which both improve visual function and halt/markedly slow retinal degeneration. Substantial challenges remain however, particularly for patients with severe visual loss from birth where normal visual pathway function has never been established, with intervention likely needed very early in infancy to fully address any potential cortical limitations and thereby optimise outcomes.
