# Correction: *Leber congenital amaurosis/early-onset severe retinal dystrophy: clinical features, molecular genetics and therapeutic interventions*

**DOI:** 10.1136/bjophthalmol-2016-309975corr1

**Published:** 2019-05-28

Kumaran N, Moore AT, Weleber RG, *et al*. Leber congenital amaurosis/early-onset severe retinal dystrophy: clinical features, molecular genetics and therapeutic interventions. *Br J Ophthalmol* 2017;101:1147–54. doi: 10.1136/bjophthalmol-2016-309975.

The authors wish to correct the legend of table 1. It reads: *Genes associated with EOSRD. †Genes more frequently associated with LCA. However it should read: †Genes associated with EOSRD. *Genes more frequently associated with LCA.

